# Role of Vascular Smooth Muscle PPARγ in Regulating AT_1_ Receptor Signaling and Angiotensin II-Dependent Hypertension

**DOI:** 10.1371/journal.pone.0103786

**Published:** 2014-08-14

**Authors:** Maria Alicia Carrillo-Sepulveda, Henry L. Keen, Deborah R. Davis, Justin L. Grobe, Curt D. Sigmund

**Affiliations:** Department of Pharmacology and Roy J. and A. Lucille Carver College of Medicine, University of Iowa, Iowa City, Iowa, United States of America; Universtiy of Maryland Schoool of Medicine, United States of America

## Abstract

Peroxisome proliferator activated receptor γ (PPARγ) has been reported to play a protective role in the vasculature; however, the underlying mechanisms involved are not entirely known. We previously showed that vascular smooth muscle-specific overexpression of a dominant negative human PPARγ mutation in mice (S-P467L) leads to enhanced myogenic tone and increased angiotensin-II-dependent vasoconstriction. S-P467L mice also exhibit increased arterial blood pressure. Here we tested the hypotheses that a) mesenteric smooth muscle cells isolated from S-P467L mice exhibit enhanced angiotensin-II AT_1_ receptor signaling, and b) the increased arterial pressure of S-P467L mice is angiotensin-II AT_1_ receptor dependent. Phosphorylation of mitogen-activated protein/extracellular signal-regulated kinase (ERK1/2) was robustly increased in mesenteric artery smooth muscle cell cultures from S-P467L in response to angiotensin-II. The increase in ERK1/2 activation by angiotensin-II was blocked by losartan, a blocker of AT_1_ receptors. Angiotensin-II-induced ERK1/2 activation was also blocked by Tempol, a scavenger of reactive oxygen species, and correlated with increased Nox4 protein expression. To investigate whether endogenous renin-angiotensin system activity contributes to the elevated arterial pressure in S-P467L, non-transgenic and S-P467L mice were treated with the AT_1_ receptor blocker, losartan (30 mg/kg per day), for 14-days and arterial pressure was assessed by radiotelemetry. At baseline S-P467L mice showed a significant increase of systolic arterial pressure (142.0±10.2 vs 129.1±3.0 mmHg, p<0.05). Treatment with losartan lowered systolic arterial pressure in S-P467L (132.2±6.9 mmHg) to a level similar to untreated non-transgenic mice. Losartan also lowered arterial pressure in non-transgenic (113.0±3.9 mmHg) mice, such that there was no difference in the losartan-induced depressor response between groups (−13.53±1.39 in S-P467L vs −16.16±3.14 mmHg in non-transgenic). Our results suggest that interference with PPAR**γ** in smooth muscle: a) causes enhanced angiotensin-II AT_1_ receptor-mediated ERK1/2 activation in resistance vessels, b) and may elevate arterial pressure through both angiotensin-II AT_1_ receptor-dependent and -independent mechanisms.

## Introduction

Peroxisome Proliferator-Activated Receptor γ (PPARγ) is a ligand-activated transcription factor belonging to the nuclear receptor superfamily which is well-known for its role in adipogenesis, lipid metabolism and glucose homeostasis (reviewed in [1]). Thiazolidinediones (TZD) are high affinity synthetic agonists for PPARγ that improve glycemic control in type 2 diabetes, and have been reported to improve vascular function and to lower arterial pressure [2] (reviewed in [3]). Mutations in PPARγ cause lipodystrophies, and in some severe cases, causes early onset insulin resistance, type 2 diabetes and hypertension [4], [5]. Mice carrying the equivalent mutation in endogenous PPARγ exhibit hypertension [6], [7] and when bred onto the leptin-deficient genetic background exhibit insulin resistance and metabolic dysfunction [8].

Genetic knock-down of PPARγ in animals confirmed a protective role of PPARγ in both endothelium and vascular muscle, however, the molecular mechanisms by which PPARγ regulates blood pressure and improves vascular function has not been completely elucidated [9]–[13]. To address this shortcoming, we developed novel models expressing dominant negative mutations in PPARγ, the same mutations that cause hypertension in human patients, selectively in endothelium and vascular smooth muscle [7], [14]–[17]. We validated that these mutations cause a gene expression profile opposite that of a PPARγ agonist consistent with their dominant negative action [18], [19].

In endothelium, interference with PPARγ caused endothelial dysfunction in response to a high fat diet which was improved by scavenging reactive oxygen species (ROS) [14]. Our data were consistent with the hypothesis that high fat diet induced the production of a PPARγ ligand which activates a protective anti-oxidant gene expression program in the endothelium. This protective mechanism cannot be induced in the presence of dominant negative PPARγ mutants. In vascular muscle, PPARγ interference causes hypertension under baseline conditions and dysfunction of both conduit and resistance blood vessels [15]. Thus, these mice faithfully phenocopy the hypertension portion of the overall phenotype exhibited by patients with identical PPARγ mutations. Mechanistically, aorta from mice with smooth muscle-specific expression of dominant negative PPARγ (S-P467L) are resistant to endogenous and exogenous nitric oxide (NO) which is reversed by Rho kinase blockade [17]. Second order mesenteric branches from these mice exhibit increased myogenic tone which is reversed by protein kinase C (PKC) inhibition [16]. They also exhibit augmented angiotensin-II (Ang-II) mediated vasoconstriction because of decreased expression of the PPARγ target gene RGS5. The present study aimed to determine whether activation of Ang-II signaling in vascular smooth muscle cells (SMC) from mesenteric arteries contributes to hypertension in S-P467L mice.

## Materials and Methods

### Animals

Transgenic mice were generated by expressing the protein coding sequence of human PPARγ carrying the P467L mutation under the control of the smooth muscle myosin heavy chain promoter (S-P467L) as described previously [15], [17]. Non-transgenic (NT) littermates were utilized as controls. All mice were continuously backcross bred to C57BL/6J mice. Mice were maintained in a 12 hour light-dark cycle (6 AM to 6 PM), fed normal rodent chow (Teklad 7013), and had access to water *ad libitum*. *In vivo* and *in vitro* experiments were performed using male mice that were four to five months of age. All of the experimental protocols were approved by the University of Iowa Animal Care and Use Committee and conducted in accordance with the National Institutes of Health Guide for the Care and Use of Laboratory Animals.

### Primary Mesenteric SMC Culture

Vascular SMCs were isolated from second and third order mesenteric arteries of NT-littermates and S-P467L mice by the explant method [20]. Briefly, mesenteric beds were cleaned of adipose and connective tissue and placed in the culture dish and maintained with Dulbecco Modified Eagle’s Medium (DMEM) containing 10% fetal bovine serum (FBS) and antibiotics and maintained in an incubator at 37°C in a humidified 5% CO_2_ atmosphere. After 72h, once VSMCs migrated from the edges of the explants, the mesenteric arteries were removed and discarded. Mesenteric SMCs exhibited the typical “hill and valley” growth morphology and were confirmed positive (>95%) for smooth muscle α-actin (Sigma) and calponin (Santa Cruz sc-28545) immunostaining, and negative for Pecam-1 (Abcam ab28364) band CD90/Thy1 (Santa Cruz sc9163). Cells at passage 3 maintain their contractile phenotype and were used in all experiments. At 80% confluence, the culture medium was replaced with serum-free medium for 24 hours to render the cells quiescent. Cells were then treated with Ang-II (0.01 µM) for 2, 5, 10, 30 and 60 minutes for determination of ERK1/2 activation and for 24, 48 and 72 hours for analysis of proliferation (as measured by PCNA expression). In some experiments mesenteric SMC were pre-incubated with losartan (1 µM) or Tempol (5 mM) for 30 min following treatment with Ang-II.

### Immunoblotting

After treatment with Ang-II, proteins were extracted from mesenteric SMC and separated by standard SDS-PAGE. Proteins (10 µg) were transferred to PVDF membranes and immunoblotted with the following primary antibodies: calponin (Santa Cruz sc-28545), ERK1/2 Cell Signaling 9102), phosphorylated ERK1/2 (Cell Signaling 9101), PCNA (Santa Cruz Sc56, Nox4 (Epitomics 3174-1), β-actin (Santa Cruz sc-130656), GAPDH (Santa Cruz: sc-32233), and their respective secondary antibodies. Proteins were detected using chemiluminescence (ECL Plus, Amersham Biosciences). Protein expression was normalized to β-actin or GAPDH.

### Gene Expression

NADPH oxidase (Nox) gene expression was evaluated from a microarray dataset of mesenteric artery of S-P467L and control mice previously reported by us (NCBI Accession Number GSE36482) [16]. Total RNA was isolated from mesenteric artery samples using Trizol Reagent (Invitrogen Life Technologies Co., Burlington, ON) and RNeasy spin columns (QIAGEN), and was quantified by Nanodrop. cDNA was synthesized from 400 ng of total RNA by RT-PCR using SuperScript III (Invitrogen). Quantitative real-time PCR (qPCR) was performed using TaqMan Fast Advanced Master Mix (Applied Biosystems), TaqMan gene expression assays (Nox4: Mn00479246_m1) and 10 ng of cDNA. ΔΔCT was calculated using GAPDH as the reference gene to determine relative mRNA expression.

### Blood Pressure and Heart Rate Measurements

Blood pressure and heart rate were measured by radiotelemetry as previously described in male mice that were four to five months of age [21], [22]. Under anesthesia with ketamine/xylazine (85.5 mg/kg and 12.5 mg/kg, respectively), a radiotelemetry probe (TA11PA-C10, Data Sciences International) was inserted in the left common carotid artery and the body of the transmitter was secured subcutaneously in the abdomen. Normal body temperature was maintained with a heating pad during intraoperative and postoperative care. After a 7-day recovery from transmitter implantation, baseline measurements were recorded for 10 seconds every 5 minutes over a 7-day period. Subsequently, mice were treated with losartan (30 mg/kg/day for 14 days) using ALZET osmotic mini-pumps (model 1002; Durect Corporation, Cupertino, CA) placed subcutaneously. Blood pressure and heart rate measurements were recorded over the last 5 days of losartan/vehicle control treatment and the data from the final 2 days is presented. Control mice received saline by osmotic mini-pumps following the time-course of losartan treatment.

### Statistical Analysis

Results are expressed as Mean±SEM and analyzed with 1 or 2-way repeated measures ANOVA followed by Tukey’s or Bonferroni multiple-comparison procedures. Student’s *t*-test was used when appropriate. P<0.05 was considered statistically significant. SigmaStat was used for all analytical comparisons.

## Results

**Figure 1:**
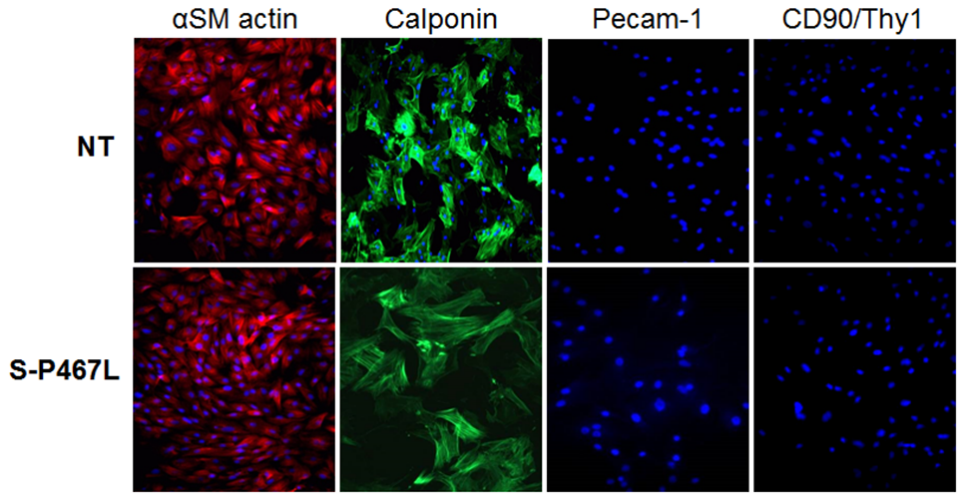
Mesenteric vascular smooth muscle culture. Characterization of mesenteric SMC cultures from NT control (upper panel) and S-P467L (bottom panel) mice. Positive immunostaining for α-actin SM (Red) and calponin (Green). Nuclei are stained with DAPI (blue). Endothelial cell contamination was excluded by negative immunostaining for Pecam-1 and CD90/Thy1. Photographed at 10X magnification.

**Figure 2:**
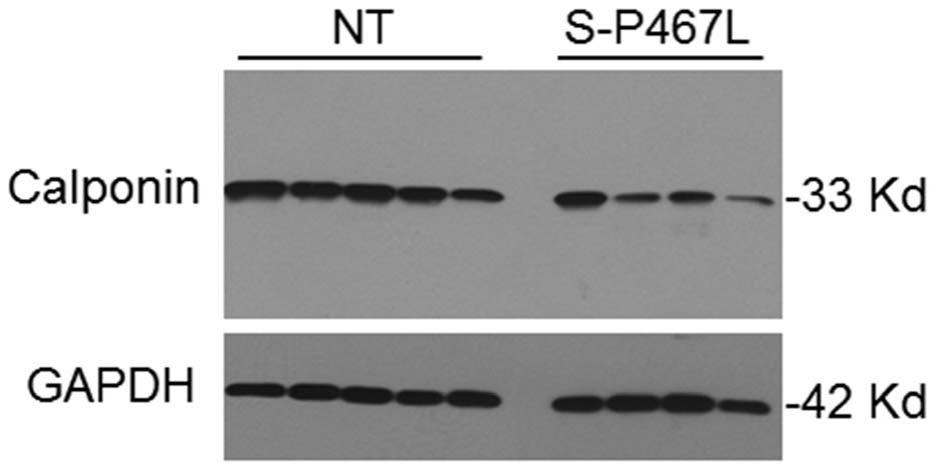
Calponin Protein Expression. Western blots showing calponin and GAPDH in mesenteric arteries from S-P467L (n = 4) and NT control mice (n = 5). The molecular weight of the indicated band is based on comparison to size markers.

**Figure 3:**
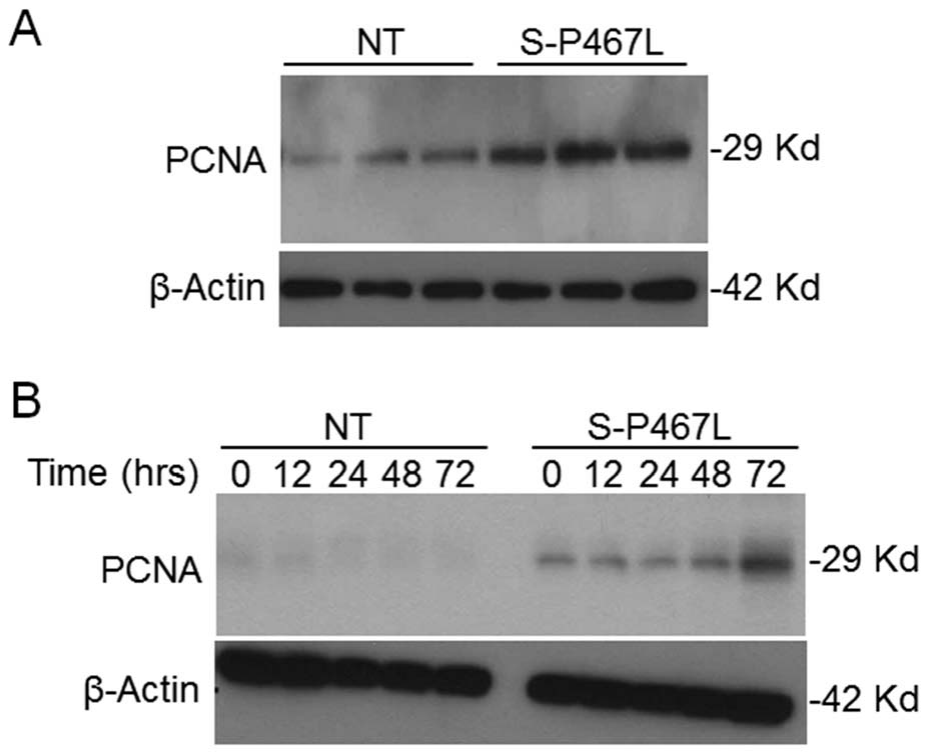
Ang-II increased PCNA in mesenteric SMC from P-467L mice. A) Western blots showing PCNA and β-Actin in cultured mesenteric SMC from S-P467L and NT mice. B) Western blots showing PCNA in response to Ang-II in mesenteric VSMC from S-P467L and NT control mice. Time (hours) after Ang-II-treatment is indicated. The molecular weight of the indicated band is based on comparison to size markers.

We first characterized mesenteric SMC primary cultures from S-P467L. The cultured cells were confirmed to be vascular smooth muscle by positive staining with α-actin and calponin, typical markers of differentiated contractile vascular SMCs (Figure 1). We also established the purity of these cultures by the absence of staining for Pecam-1 and CD90/Thy1, markers for endothelial cells. The apparent decrease in calponin immunostaining in cultured cells from S-P467L mice was consistent with a decrease in calponin expression in mesenteric arteries from those mice (Figure 2). The decrease in a classical vascular SMC marker suggested the cells may have undergone a more rapid change from a contractile phenotype to a synthetic or proliferative phenotype. Consistent with this, mesenteric SMCs from S-P467L showed increased expression of proliferating cell nuclear antigen (PCNA) protein compared to mesenteric SMC from control non-transgenic (NT) mice under basal conditions (Figure 3A). In addition, stimulation with Ang-II (0.01 µM) for 72 hours increased PCNA protein expression by approximately 2-fold in vascular SMCs from S-P467L, but not from NT control mice (Figure 3B).

**Figure 4:**
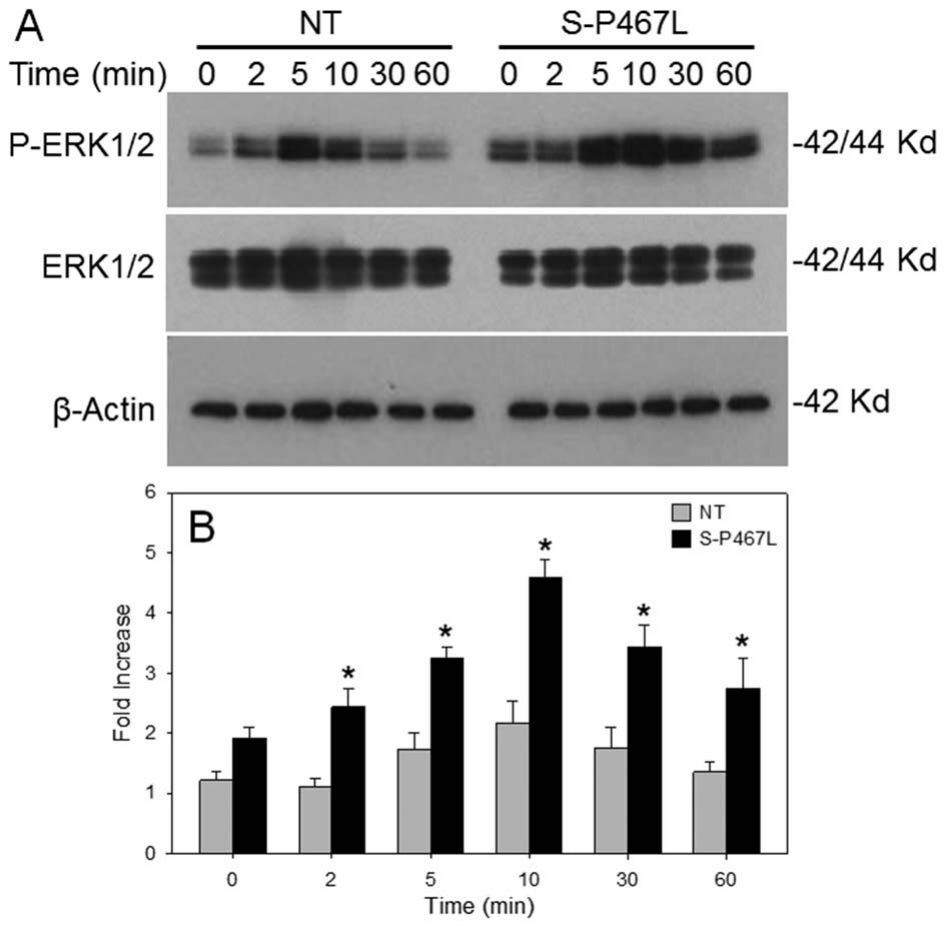
ERK1/2 activation. A) Western blots showing phosphorylated and total ERK1/2 and β-actin at baseline and in response to Ang-II in mesenteric SMC from S-P467L and control mice. Time (minutes) is indicated. B) Quantification of 4 independent experiments is shown in the graph. *, P<0.05 times 2–60 minutes vs 0, and S-P4567L vs NT controls.

**Figure 5:**
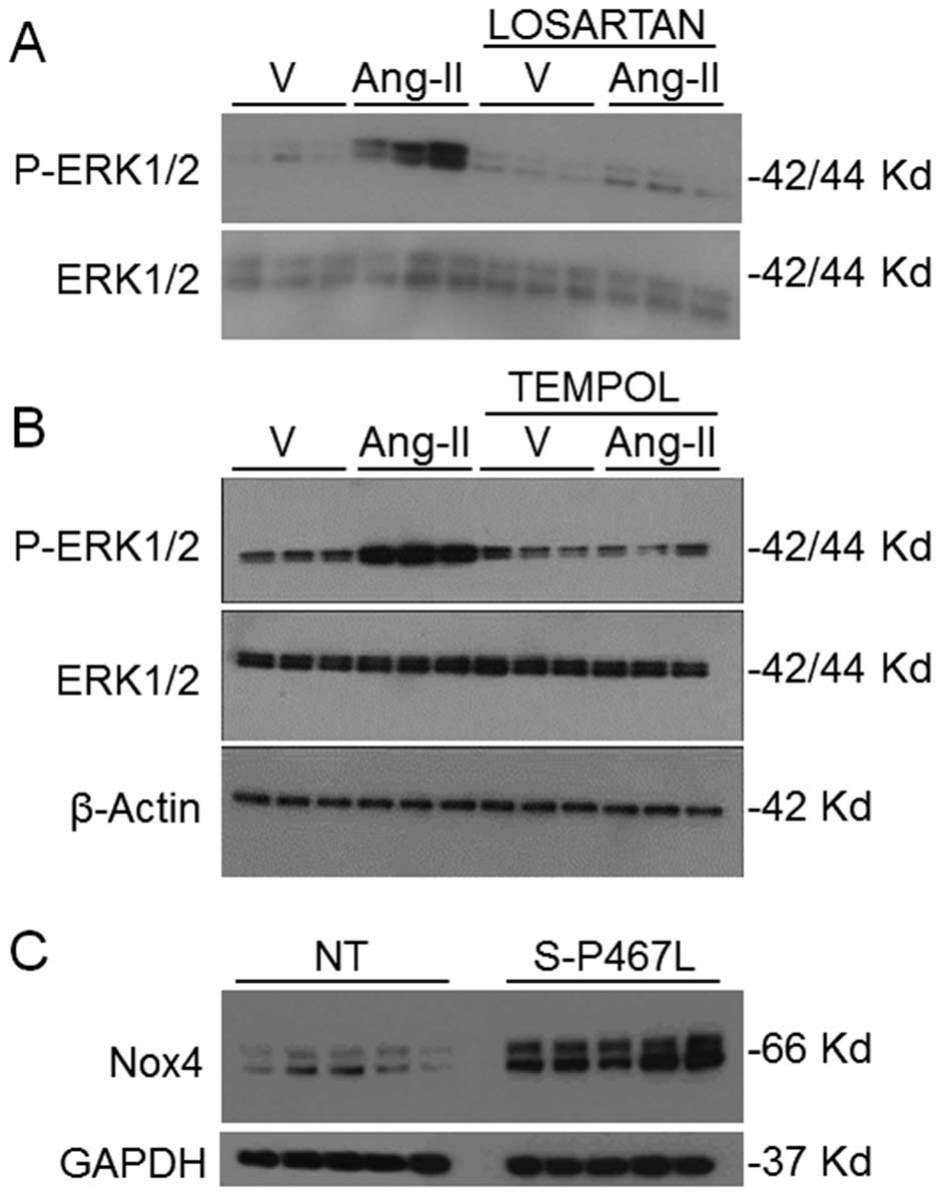
ERK1/2 activation is AT_1_ receptor and redox sensitive. Western blots showing phosphorylated and total ERK1/2 and β-actin in response to Ang-II pretreated with 1 µM Losartan (A) or 5 mM Tempol (B) in mesenteric vascular SMC from S-P467L mice. C) Western blots showing Nox4 and GAPDH in mesenteric SMC from S-P467L and control (NT) mice. V, vehicle; Ang-II, angiotensin-II.

We next tested the hypothesis that Ang-II-treated mesenteric SMC from S-P467L mice have augmented ERK1/2 activation. At baseline, there was a trend for increased activation of ERK1/2 in mesenteric SMCs from S-P467L compared to NT control cells (Figure 4). Mesenteric SMCs from S-P467L showed a greater phosphorylation of ERK1/2 than NT control cells in response to Ang-II. Ang II-induced ERK1/2 phosphorylation was maintained for 60 min in mesenteric SMCs from S-P467L, while 10 minutes was the maximum period of ERK1/2 phosphorylation in mesenteric SMCs from NT mice. There was a slight decrease in baseline phosphorylated JNK without a change in total JNK, but no increase in phosphorylated JNK in response to Ang-II (data not shown). The increase in ERK1/2 activation by Ang-II was AT_1_ receptor-mediated as ERK1/2 phosphorylation could be blocked by losartan (Figure 5A). The Ang-II-mediated activation of ERK1/2 in vascular SMCs from S-P467L was abolished in the presence of Tempol, a scavenger of superoxide suggesting this is redox mediated (Figure 5B). Examination of microarray data of mesenteric artery RNA from S-P467L and control mice revealed no changes in the expression of Nox1 or Nox3 [16]. Expression of Nox2 was increased by 20%, whereas expression of Nox4 was decreased by 50%. Quantitative RT-PCR confirmed decreased expression of Nox4 in mesenteric artery of S-P467L (to 0.45±0.08 of control levels, P<0.02). Despite a decrease in Nox4 mRNA, there was evidence of increased Nox4 protein in mesenteric vessels from S-P467L mice (Figure 5C).

**Figure 6:**
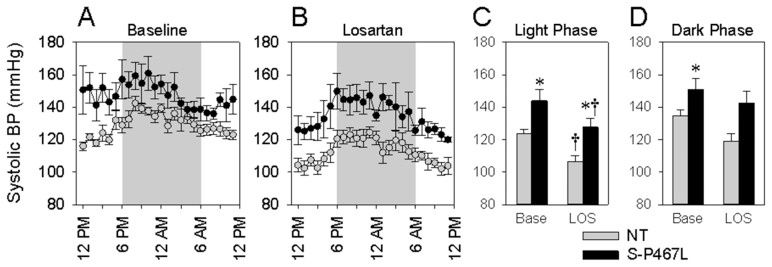
Blood pressure responses to losartan treatment. Hourly average radiotelemetric systolic arterial pressure recordings from S-P467L (filled) and control (grey) mice at baseline (A) and in response to losartan (B). Average blood pressure in the light (C) and dark (D) phase. Data from losartan-treated mice were collected from the last 2 days of losartan (LOS) treatment. All data are mean ± SEM. *, P<0.05 vs NT; ^†^, P<0.05 vs baseline.

We previously reported that S-P467L mice exhibit a pressor response to acute Ang-II infusion that is similar to control mice [16]. We therefore tested whether augmented mesenteric SMC Ang-II signaling contributes to the chronically elevated blood pressure in S-P467L mice. As we showed previously, S-P467L mice exhibited increased baseline arterial pressure compared with littermate controls (Figure 6A, C). We treated mice with losartan to determine if endogenous RAS signaling contributes to the increased arterial pressure. Losartan significantly lowered arterial pressure in S-P467L mice (Figure 6B–D) to the level observed in untreated littermate controls. Losartan also lowered arterial pressure in control mice, but the change in arterial pressure in response to losartan was equal in both groups (S-P467L: −17.2±12.9 mmHg; NT: −16.4±4.2 mmHg), although the variability in the S-P467L group was larger. Interestingly, whereas the heart rate in control mice modestly declined in response to losartan (−11.5±15.6 beats/min), this did not occur in S-P467L mice (+1.7±9.2 beats/min).

## Discussion

Studies in animals and human subjects have convincingly demonstrated that PPARγ plays a crucial role in the regulation of vascular tone and control of arterial blood pressure [6],[23],[24]. The strongest evidence supporting a role for PPARγ in arterial pressure regulation comes from genetic studies where patients bearing rare dominant negative mutations in PPARγ exhibit severe early onset hypertension [4]. This is further supported by a recent report identifying two new mutations in PPARγ that are associated with increased arterial pressure due to aberrant activation of the renin-angiotensin system [5], [25]. The link between PPARγ and the renin-angiotensin system is further supported by our studies showing that mice expressing the P467L dominant negative mutation in PPARγ specifically in vascular smooth muscle (S-P467L mice) caused increased contraction of mesenteric artery to Ang-II [16]. Thus, the rationale for the current study was to explore the role of Ang-II-dependent signaling and arterial pressure regulation. The main findings of the current study are that 1) mesenteric SMC from S-P467L mice exhibit increased Ang-II ERK1/2 signaling through an AT_1_ receptor-dependent and oxidant stress-dependent mechanism, and 2) the increased baseline blood pressure observed in S-P467L mice is lowered to normal levels by short-term AT_1_ receptor blockade.

Mesenteric SMC from S-P467L mice exhibited increased proliferation, as measured by increased expression of PCNA, a marker of proliferation, and decreased expression of calponin, a contractile marker. These data suggest that the mesenteric SMC from S-P467L mice may be undergoing phenotypic modulation, that is, a shift from a contractile state to a proliferative state. It has been reported that vascular SMC PPARγ prevents the switch from a contractile to a proliferative state during vascular injury and hypertension [26], [27]. Similarly, SMC derived from mice carrying a gene targeted mutation in PPARγ equivalent to the P467L mutation employed herein exhibited increased proliferation [28]. It is therefore not surprising that Ang-II further increased PCNA expression in mesenteric SMC from S-P467L. These data suggest that loss of PPARγ function in vascular SMC promotes a proliferative phenotype. This is supported by studies showing that PPARγ has a vascular protective effect in Ang-II-induced vascular remodeling [13], [23].

Mesenteric SMC from S-P467L mice exhibited augmented responsiveness to Ang-II as measured by a rapid and potent induction of ERK1/2 phosphorylation. This effect was abolished in the presence of Tempol, suggesting it is mediated by increased oxidative stress. It is now well accepted that NADPH oxidase-derived ROS is one of the main mediators of Ang-II-induced vascular dysfunction [29], [30]. We evaluated the expression of NADPH oxidase isoforms in our model and Nox4 was the only one found to be significantly altered. Although Nox4 mRNA levels were decreased in mesenteric arteries from S-P467L mice, Nox4 protein was markedly increased. Interestingly, increased Nox4 was shown to correlate with decreased expression of PPARγ in human pulmonary artery endothelial and smooth muscle cells, and rosiglitazone, a potent PPARγ agonist blunts the increase in Nox4 expression induced by hypoxia in mouse lung [31]. Hydrogen peroxide, a product of Nox4 activity, has been demonstrated to act as a proliferative factor in VSMC [32], and decreases PPARγ expression and activity in endothelial cells [33]. At face value, these results suggest that Nox4 may be a mediator of increased proliferation and AT_1_ receptor signaling in SMC from S-P467L mice. However, that vascular dysfunction and increased agonist-induced contraction in aorta, and increased myogenic tone in mesenteric artery was not reversed by Tempol *in vivo* makes it unlikely that Nox4 mediates the vascular dysfunction or hypertension observed in S-P467L mice [16], [17]. Of course, we cannot rule out that Nox4 may have a local effect in SMC that indirectly contributes over the long term to vascular dysfunction in S-P467L. Further studies would be needed to clarify its function.

Future studies should also examine the importance of inflammation. There is a reciprocal relationship between PPARγ and AT_1_ receptors. Just as PPARγ regulates AT_1_ receptor expression, Ang-II also down-regulates PPARγ in aorta, and this promotes inflammation [34]. We have previously reported that S-P467L mice exhibit increased expression of pro-inflammatory and pro-atherogenic genes particularly when bred on the ApoE-deficient genetic background [35].

We considered two possible mechanisms accounting for the increase in AT_1_ receptor signaling. The first is that PPARγ interference caused increased expression of AT_1_ receptor mRNA. In support of this, PPARγ activation decreases expression of the Ang-II AT_1_ receptor in smooth muscle [36] and cardiac fibroblasts [37], and PPARγ mutations increase expression of genes in the renin-angiotensin system including renin, angiotensinogen and AT_1_ receptor [5]. However, we reported that AT_1_ receptor mRNA was unchanged in mesenteric artery of S-P467L mice [16]. Alternatively, PPARγ-interference can increase activity of the AT_1_ receptor signaling complex. PPARγ activation represses Ang-II signaling in IgA nephropathy [38] and attenuates Ang-II-induced inflammation in vascular smooth muscle cells [39]. We previously showed that PPARγ-interference results in decreased expression of regulator of G protein signaling 5 (RGS5) mRNA in mesenteric artery from S-P467L mice [16]. RGS5 is a novel PPARγ target gene, and it’s decreased expression was responsible for augmented myogenic tone and Ang-II-mediated contraction of mesenteric artery. Consequently, the available evidence suggests that interference with PPARγ increases AT_1_ receptor signaling as a result of increased post-receptor activation and not due to increased expression of AT_1_ receptor mRNA.

PPARγ activation lowers blood pressure in models of Ang-II infusion [23], and in Ang-II-dependent hypertensive transgenic mice [24]. To evaluate whether augmented Ang-II signaling in vascular SMC contributes to the hypertensive phenotype found in S-P467L mice, we treated S-P467L mice with the AT_1_ receptor antagonist losartan. Losartan lowered blood pressure in S-P467L to the level observed in untreated NT control mice. The fact that blood pressure was lowered to control levels by losartan would be consistent with the observation that AT_1_ receptor blockers were said to be effective antihypertensives in patients with PPARγ mutations leading to activation of the renin-angiotensin system [5]. These patients carry PPARγ mutations in all cells whereas in S-P467L, the mutation is restricted to vascular smooth muscle. This suggests that the loss of PPARγ signaling in smooth muscle may be sufficient to phenocopy the hypertension that results from the presence of the mutation in all cells in human patients. That losartan normalizes blood pressure in S-P467L mice would also be consistent with our observation that there is a large increase in contraction of mesenteric arteries to Ang-II [16]. However, it is important to recognize that the losartan-induced depressor response was similar in S-P467L and in control mice. This observation would be consistent with other data showing that acute Ang-II infusion equivalently increased blood pressure in S-P467L and control mice [16]. We cannot rule out that the precise mechanisms of the losartan-induced depressor response is different in S-P467L mice, and that S-P467L and NT mice may exhibit subtly different Ang-II-dependent mechanisms regulating arterial pressure. This may be evidenced by the differential effects of losartan on heart rate. NT mice exhibited a modest bradycardia whereas there was no change in heart rate in S-P467L. We have recently reported that S-P467L exhibit autonomic dysfunction which as we show here may be preserved after AT_1_ receptor blockade [40]. We also cannot rule out that other mechanisms serve to chronically regulate blood pressure in the S-P467L model. Indeed, we previously reported that unlike mesenteric arteries, contraction of the aorta is augmented in response to RhoA/Rho kinase agonists and that arterial blood pressure in S-P467L mice is markedly decreased after infusion of a Rho kinase inhibitor [17].
